# Admission Levels of Interleukin 10 and Amyloid β 1–40 Improve the Outcome Prediction Performance of the Helsinki Computed Tomography Score in Traumatic Brain Injury

**DOI:** 10.3389/fneur.2020.549527

**Published:** 2020-10-30

**Authors:** Jussi P. Posti, Riikka S. K. Takala, Rahul Raj, Teemu M. Luoto, Leire Azurmendi, Linnéa Lagerstedt, Mehrbod Mohammadian, Iftakher Hossain, Jessica Gill, Janek Frantzén, Mark van Gils, Peter J. Hutchinson, Ari J. Katila, Pia Koivikko, Henna-Riikka Maanpää, David K. Menon, Virginia F. Newcombe, Jussi Tallus, Kaj Blennow, Olli Tenovuo, Henrik Zetterberg, Jean-Charles Sanchez

**Affiliations:** ^1^Clinical Neurosciences, Department of Neurosurgery, Turku Brain Injury Centre, Turku University Hospital, University of Turku, Turku, Finland; ^2^Perioperative Services, Intensive Care Medicine and Pain Management, Department of Anesthesiology and Intensive Care, Turku University Hospital, University of Turku, Turku, Finland; ^3^Department of Neurosurgery, Helsinki University Hospital, University of Helsinki, Helsinki, Finland; ^4^Department of Neurosurgery, Tampere University Hospital, Tampere University, Tampere, Finland; ^5^Department of Specialities of Internal Medicine, Faculty of Medicine, University of Geneva, Geneva, Switzerland; ^6^Turku Brain Injury Centre, Turku University Hospital, University of Turku, Turku, Finland; ^7^Neurosurgery Unit, Department of Clinical Neurosciences, Addenbrooke's Hospital, University of Cambridge, Cambridge, United Kingdom; ^8^National Institute of Nursing Research, National Institutes of Health, Bethesda, MD, United States; ^9^VTT Technical Research Centre of Finland Ltd., Tampere, Finland; ^10^Division of Anaesthesia, Addenbrooke's Hospital, University of Cambridge, Cambridge, United Kingdom; ^11^Department of Psychiatry and Neurochemistry, Institute of Neuroscience and Physiology, The Sahlgrenska Academy, University of Gothenburg, Gothenburg, Sweden; ^12^Clinical Neurochemistry Laboratory, Sahlgrenska University Hospital, Gothenburg, Sweden; ^13^Department of Neurodegenerative Disease, University College London Institute of Neurology, London, United Kingdom; ^14^The United Kingdom Dementia Research Institute at University College London, University College London, London, United Kingdom

**Keywords:** traumatic brain injury, biomarkers, outcome prediction, Helsinki CT score, interleukin 10 (IL10), beta amyloid 1–40, panel analysis

## Abstract

**Background:** Blood biomarkers may enhance outcome prediction performance of head computed tomography scores in traumatic brain injury (TBI).

**Objective:** To investigate whether admission levels of eight different protein biomarkers can improve the outcome prediction performance of the Helsinki computed tomography score (HCTS) without clinical covariates in TBI.

**Materials and methods:** Eighty-two patients with computed tomography positive TBIs were included in this study. Plasma levels of β-amyloid isoforms 1–40 (Aβ40) and 1–42 (Aβ42), glial fibrillary acidic protein, heart fatty acid-binding protein, interleukin 10 (IL-10), neurofilament light, S100 calcium-binding protein B, and total tau were measured within 24 h from admission. The patients were divided into favorable (Glasgow Outcome Scale—Extended 5–8, *n* = 49) and unfavorable (Glasgow Outcome Scale—Extended 1–4, *n* = 33) groups. The outcome was assessed 6–12 months after injury. An optimal predictive panel was investigated with the sensitivity set at 90–100%.

**Results:** The HCTS alone yielded a sensitivity of 97.0% (95% CI: 90.9–100) and specificity of 22.4% (95% CI: 10.2–32.7) and partial area under the curve of the receiver operating characteristic of 2.5% (95% CI: 1.1–4.7), in discriminating patients with favorable and unfavorable outcomes. The threshold to detect a patient with unfavorable outcome was an HCTS > 1. The three best individually performing biomarkers in outcome prediction were Aβ40, Aβ42, and neurofilament light. The optimal panel included IL-10, Aβ40, and the HCTS reaching a partial area under the curve of the receiver operating characteristic of 3.4% (95% CI: 1.7–6.2) with a sensitivity of 90.9% (95% CI: 81.8–100) and specificity of 59.2% (95% CI: 40.8–69.4).

**Conclusion:** Admission plasma levels of IL-10 and Aβ40 significantly improve the prognostication ability of the HCTS after TBI.

## Introduction

Traumatic brain injury (TBI) is a highly heterogeneous disease (1) and a leading cause of long-term disability globally (2). It is clear that outcome after TBI solely does not depend only on the given care in the acute and late phases, but also on the injury type and severity, patient's clinical characteristics, and eventual brain tissue fate (3, 4). Improved outcome models may help better stratify patients for different treatment and monitoring strategies and provide information about expected gross outcomes to clinicians, patients, and their families.

TBI is classically divided into mild, moderate, and severe based on the initial assessment using the Glasgow Coma Scale (GCS) score upon admission (5). The GCS score is one of the strongest clinical outcome predictors (3) but does not consider the complex pathophysiological characteristics of TBI. Furthermore, GCS assessment may be confounded by subjective interrater variability and patient's intoxication or sedation (6, 7).

Early structural intracranial abnormalities detected on head computed tomography (CT) have been suggested as complementary or independent outcome predictors. The Marshall CT classification (8) was not originally designed to be an outcome measure tool, but its features have been successfully incorporated into the International Mission for Prognosis and Analysis of Clinical Trials in TBI (IMPACT) (9) and the Corticosteroid Randomization After Significant Head injury (10) prognostication models, which have been comprehensively validated (11). After the Marshall CT classification, outcome prediction-weighted CT classifications have emerged. Rotterdam CT score (12), Helsinki CT Score (HCTS), (13) and Stockholm CT score (14) have shown promise in prognostication of patients with CT-positive findings. The latter two reportedly provide more information on the structural pathology and more accurate outcome prediction than earlier models (15).

Several brain-enriched protein biomarkers have been studied in combination and isolation as tools for predicting TBIs of different severities (16–18). Biomarkers may offer incremental value in outcome prediction when used in combination with neuroimaging scores. We recently studied eight biomarkers [β-amyloid isoforms 1–40 [Aβ40] and 1–42 [Aβ42], glial fibrillary acidic protein [GFAP], heart fatty acid-binding protein [H-FABP], interleukin 10 [IL-10], neurofilament light chain [NF-L], S100 calcium-binding protein B [S100B], and total tau [t-tau]] and their ability to discriminate CT-negative and CT-positive patients with TBIs of different severities. We found that panels of biomarkers significantly outperformed individual biomarkers in this setting (19).

The overall aim of this study was to see whether the biomarkers listed earlier improved the prediction of outcome using an admission head CT score. As these biomarkers are of different cellular origins, we planned to investigate each separately as well as combined. The HCTS was chosen due to its ability to be reliably implemented, and it has an extensive validation background (15, 20–23). We hypothesized that the prognostic performance of the HCTS would improve after adding blood-based biomarkers.

## Methods

### Study Population and Clinical Characteristics

This prospective study was part of the European Union-funded TBIcare (Evidence-Based Diagnostic and Treatment Planning Solution for Traumatic Brain Injuries) project, where we recruited patients with acute TBIs at the Turku University Hospital, Finland, from November 2011 to October 2013. All patients were treated according to the local protocols based on existing international guidelines and recommendations at that time (24).

The total available cohort of patients with head injury consisted of 620 patients. Of these, 203 patients met the following inclusion criteria: (i) age ≥ 18 years and (ii) clinical diagnosis of TBI and indications for acute head CT according to the National Institute for Health and Care Excellence criteria (25), and did not meet the following exclusion criteria: (i) blast-induced or penetrating injury, (ii) chronic subdural hematoma, (iii) inability to live independently due to a preexisting brain disease, (iv) TBI or suspected TBI not needing head CT, (v) more than 2 weeks from the injury, (vi) not living in the hospital district thereby preventing follow-up visits, (vii) not speaking the native language (Finnish), or (viii) no consent received.

In this study, we included those patients who had admission levels of plasma Aβ40, Aβ42, GFAP, H-FABP, IL-10, NF-L, S100B, and t-tau obtained within 24 h after hospital admission available (*n* = 160). From these patients, we included those who had Glasgow Outcome Scale—Extended (GOSE) scores assessed 4–16 months after injury [assessed by an experienced neurologist [OT], *n* = 137, the average time between injury and GOSE was 7.82 months, ±3.33]. Outcomes were defined as favorable (GOSE 5–8), and unfavorable (GOSE 1–4), complete recovery (GOSE 8), and incomplete recovery (GOSE <8) (17). Traditionally, the first categorization is used in terms of moderate to severe TBI and the latter in mild TBI. As the patients were not classified according to their initial GCS scores but according to their HCTS scores in the current study, we used both categorizations. The admission head CT scans were blindly evaluated by three senior neurotrauma researchers (neurosurgeons) as described later. The patients were divided into the main study cohort (CT-positive, *n* = 82, 60%) and comparison cohort (CT-negative, *n* = 55, 40%). Data on TBI-related deaths were collected up to 12 months after injury.

The GCS scores were assessed by paramedics at the scene of the accident or during transport and/or by an emergency physician at the time of admission. The lowest recorded post-resuscitation GCS was used in the demographic data (16, 26). Hypoxia was defined as any event of oxygen saturation of <90% and hypotension as any period of systolic blood pressure level of <100 mmHg in patients aged 50–69 years and <110 mmHg in patients aged 18–49 years and ≥70 years (24). Anemia was defined as a hemoglobin concentration of <100 g/L. Hypoglycemia was defined as a glucose level of <4.4 mmol/L. These thresholds were based on the latest international recommendation (24). Injury Severity Score (ISS) (27) was used to evaluate the overall injury load.

The ethical review board of the Hospital District of Southwest Finland approved the study protocol (decision 68/180/2011). All patients or their next of kin were informed about the study in both oral and written forms. Written informed consent was obtained according to the World Medical Association's Declaration of Helsinki.

### Biomarker Analyses

Blood samples for plasma Aβ40, Aβ42, GFAP, H-FABP, IL-10, NF-L, S100B, and t-tau were drawn within 24 h from admission. Plasma H-FABP and IL-10 were analyzed using the K151HTD and K151QUD kits, respectively, from Meso Scale (Meso Scale Diagnostics, Rockville, MD, USA), and S100B was measured using EZHS100B-33K kit from Millipore (Millipore, Billerica, MA, USA) according to the manufacturers' recommendations in a research laboratory in Geneva, Switzerland. The plasma levels of GFAP, NF-L, and t-tau were assessed using the Human Neurology 4-Plex A assay on an HD-1 Single molecule array (Simoa) instrument according to the instructions from the manufacturer (Quanterix, Billerica, MA, USA) in the Clinical Neurochemistry Laboratory, Sahlgrenska University Hospital, Mölndal, Sweden. Plasma Aβ40 and Aβ42 concentrations were measured using a duplex Simoa immunoassay (Quanterix, Billerica, MA, USA) in a research laboratory in Bethesda, MD, USA.

The lower limits of detection, the lower limits of quantification, and the calibration ranges for the blood-based biomarkers are shown in Supplementary Table 1. One patient had an S100B level below the lower limit of detection range, and therefore, the concentration of 1 pg/ml was applied, permitting statistical analysis. This applied concentration did not affect the statistics results. All biomarker measurements were performed by board-certified laboratory technicians who were blinded to clinical data.

### Computed Tomography Scan Grading

Three senior neurotrauma researchers (JP, RR, and TL) evaluated 137 head CT scans and classified them according to the HCTS (13). First, two researchers (JP and RR) independently and blindly analyzed the scans and coded the findings, and the third (TL) evaluated the results. Next, the third evaluated all the scans, emphasizing the cases with conflicting results provided by the two independent researchers. Last, the cases with disagreement were assessed in a joint meeting.

### Statistical Analysis

The normality of distribution of the biomarker levels was assessed with the Kolmogorov–Smirnov test and by visually inspecting histograms. The demographic data on age, sex, pupil reactivity, extracerebral injuries, events of hypoxia, events of hypotension, events of hypoglycemia, anemia, hospital admission/discharge, and outcome were normally distributed and are presented as mean ± standard deviation. Differences between groups were analyzed with *t-*tests. There were patients with missing data on pupil reactivity, events of hypoxia, hypotension, and hypoglycemia, and these were excluded from the comparative analysis. Data on GCS, ISS, (27), and HCTS sum are presented in medians and ranges. Differences between groups are analyzed with the Mann–Whitney *U-*test. The levels of the biomarkers were not normally distributed and are presented as medians with interquartile ranges (IQRs). Differences in biomarker levels between the two outcome groups were analyzed with the Mann–Whitney *U-*test.

The partial area under the curve (pAUC) of the receiver operating characteristic (ROC) was used to compare only a portion of the biomarkers AUC curves, which here was set to the clinically relevant range of 90–100% sensitivity. Panels were developed by the iterative combination of biomarkers and thresholds method using the Panelomix toolbox (28). For each biomarker, several cutoffs were selected, and the best combination of markers and thresholds was selected to give the best panel performance. The size of the panels was set to a maximum of (i) first two and then (ii) three covariates (from the pool of the biomarkers and the HCTS) and was evaluated when sensitivity was set at 90–100%. Hence, an optimal predictive panel means combining covariates that yields a set of the best specificity, sensitivity, and pAUC. *P* < 0.05 were considered significant.

The first round of the head CT scan review included reviews by RR and JP. The inter-rater reliability was assessed with Cohen's kappa statistic. The overall inter-rater reliability between the three reviewers was assessed with the intraclass correlation coefficient (two-way mixed-effects).

Excluding the Panelomix toolbox analysis, the statistical analysis was carried out using the IBM SPSS Statistics version 25 (IBM Corp, New York).

## Results

### Demographics, Computed Tomography Findings, Outcomes, and Blood Samples

**Figure 1:**
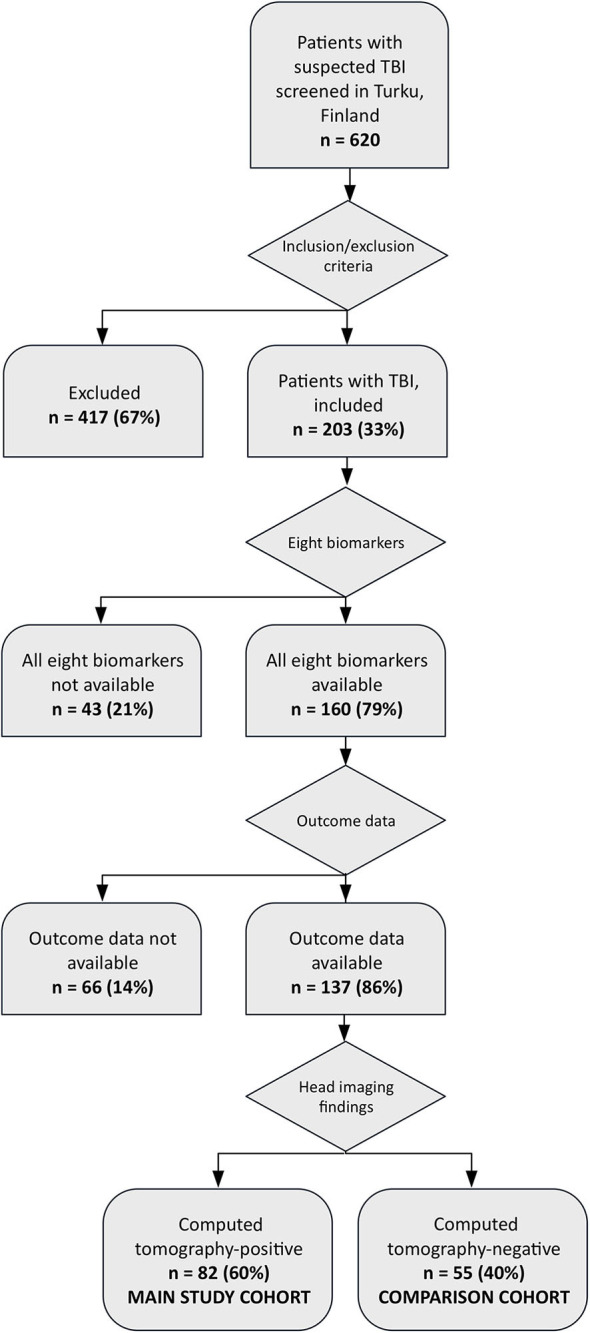
Study recruitment flow chart.

The number of eligible patients was 137. Out of these, 82 patients (60%) were CT-positive, and 55 patients (40%) were CT-negative (Figure 1). The CT-positive patients constituted the main study group. Differences in baseline characteristics between CT-positive (main study group) and CT-negative patients (comparison study group) are shown in Supplementary Material. Briefly, patients in the CT-positive group were older (mean age 50 vs. 44 years), more often male (78 vs. 62%), had lower GCS scores (median 14 vs. 15), more often abnormal pupillary light reactions (15 vs. 4%), higher ISSs (median 18 vs. 6), and less frequently had a favorable outcome (60 vs. 93%) compared with patients in the CT-negative group. The main study group differed from the total potential head injury population (*n* = 620) only in terms of sex: in the main study group, 78% were males and in the total available cohort 71%.

In the CT-positive group, the mean age was 50.5 years (SD ±20.4), 78% were male, the median GCS score was 14 and 60% had a favorable outcome. The CT-positive patients with a favorable outcome were younger, had higher GCS scores, lower ISSs, and underwent less mass lesion evacuations compared with the CT-positive patients with an unfavorable outcome (Tables 1, 2). In the CT-positive group, there were no differences in time elapsed between injury date and outcome assessment date when a patient had favorable and unfavorable outcomes (*p* = 0.584) and when complete and incomplete recovery (*p* = 0.320) were compared.

**Table 1:** Demographics of the whole study cohort—all patients.

**Variable type**	**Variable**		**Main study cohort, CT-positive** ** (*n =* 82)**	**Comparison study cohort, CT-negative** ** (*n =* 55)**	***p*-value**
Demographic	Age (years, mean ± SD)		50.46 ± 20.35	43.67 ± 18.21	**0.048**
	Sex (male/female)		64 (78%)/18 (22%)	34 (62%)/21 (38%)	**0.039**
	GCS (median [range])		14 (3–15)	15 (3–15)	**0.043**
	Pupil reactivity	Unreactive/sluggish/reactive	9 (11%)/3 (4%)/61 (74%)^a^	1 (2%)/1 (2%)/52 (95%)^b^	**0.020**
	ISS (median [range])		18 (1–50)	6 (1–57)	**0.001**
	Isolated TBI		49 (60%)	32 (59%)	0.856
	Evacuated mass lesion		24 (29%)	0 (0%)	** <0.001**
	Hypoxia		6 (7%)^c^	1 (2%)^d^	0.203
	Hypotension		3 (4%)^e^	0 (0%)^f^	0.182
	Hypoglycemia		0 (0%)^g^	0 (0%)^f^	–
	Anemia		3 (4%)	0 (0%)	0.175
	Admitted to hospital		76 (93%)	33 (60%)	** <0.001**
	Outcome	Favorable (GOSE 5–8)	49 (60%)	51 (93%)	** <0.001**
		Unfavorable (GOSE 1–4)	33 (40%)	4 (7%)	** <0.001**
		Complete (GOSE 8)	10 (12%)	23 (42%)	** <0.001**
		Incomplete (GOSE 1–7)	72 (88%)	32 (58%)	** <0.001**
	TBI-related deaths		11 (12%)	1 (2%)	** <0.001**
HCTS	Mass lesion types	Subdural hematoma	53 (65%)	–	–
		Intracerebral hematoma	53 (65%)	–	–
		Epidural hematoma	11 (13%)	–	–
	Mass lesion size >25 cm^3^		26 (32%)	–	–
	Intraventricular hemorrhage		21 (26%)	–	–
	Suprasellar cisterns	Normal	47 (57)	–	–
		Compressed	31 (38%)	–	–
		Obliterated	4 (5%)	–	–
	Sum (median [range])		4 (−3 to 14)	0	** <0.001**

*Statistically significant p-values are in bold. SD, standard deviation; GCS, Glasgow Coma Scale; ISS, Injury Severity Score; Isolated TBI, traumatic brain injury without concomitant extracerebral injuries; Hypoxia, event of hypoxia after injury; Hypotension, event of hypotension after injury; Anemia, anemia after injury; TBI, traumatic brain injury; GOSE, Glasgow Outcome Scale—Extended; HCTS, Helsinki Computed Tomography Score; CT-positive, Computed tomography-positive; CT-negative, Computed tomography-negative*.

a *Data missing on nine patients*.

b *Data missing on one patient*.

c *Data missing on seven patients.*.

d *Data missing on 11 patients.*.

e *Data missing on two patients.*.

f *Data missing on eight patients.*.

g *Data missing on three patients*.

**Table 2:** Demographics of the main study cohort—Computed tomography-positive patients divided into patients with favorable outcome (Glasgow Outcome Scale—Extended 5–8) and unfavorable outcome (Glasgow Outcome Scale—Extended 1–4).

**Variable type**	**Variable**		**Favorable outcome** ** (*n =* 49)**	**Unfavorable outcome** ** (*n =* 33)**	***p*-value**
Demographic	Age (years, mean ± SD)		44.69 ± 19.55	59.03 ± 18.67	**0.001**
	Sex (male/female)		37 (76%)/12 (24%)	27 (82%)/6 (18%)	0.505
	GCS (median [range])		14 (3–15)	9 (3–15)	**0.001**
	Pupil reactivity	Unreactive/sluggish/reactive	3 (6%)/1 (2%)/38 (78%)^a^	6 (18%)/2 (6%)/52 (70%)^b^	0.075
	ISS (median [range])		17 (1–41)	24 (6–50)	**0.001**
	Isolated TBI		29 (60%)	20 (61%)	0.889
	Evacuated mass lesion		12 (25%)	12 (36%)	** <0.001**
	Hypoxia		3 (6%)^c^	3 (9%)^d^	0.608
	Hypotension		2 (4%)	1 (3%)	0.813
	Hypoglycemia		0 (0%)^e^	0 (0%)^f^	–
	Anemia		2 (4%)	1 (3%)	0.800
	Admitted to hospital		44 (90%)	32 (98%)	0.226
	Outcome	Complete recovery (GOSE 8)	10 (20%)	0 (0%)	**0.005**
		Incomplete recovery (GOSE 1–7)	39 (80%)	33 (100%)	**0.005**
	TBI-related deaths		0 (0%)	11 (33%)	**0.001**
HCTS	Mass lesion types	Subdural hematoma	27 (55%)	26 (79%)	**0.028**
		Intracerebral hematoma	27 (55%)	26 (79%)	**0.028**
		Epidural hematoma	7 (14%)	4 (12%)	0.781
	Mass lesion size >25 cm^3^		10 (20%)	16 (49%)	**0.007**
	Intraventricular hemorrhage		9 (18%)	12 (36%)	0.069
	Suprasellar cisterns	Normal	33 (67%)	14 (42%)	** <0.001**
		Compressed	13 (27%)	18 (54%)	**0.010**
		Obliterated	3 (6%)	1 (3%)	**0.031**
	Sum (median [range])		3 (−3 to 14)	5 (0–10)	**0.004**

*Statistically significant p-values are in bold. SD, standard deviation; GCS, Glasgow Coma Scale; ISS, Injury Severity Score; Isolated TBI, traumatic brain injury without concomitant extracerebral injuries; Hypoxia, event of hypoxia after injury; Hypotension, event of hypotension after injury; Anemia, anemia after injury; TBI, traumatic brain injury; GOSE, Glasgow Outcome Scale—Extended; HCTS, Helsinki Computed Tomography Score*.

a *Data missing on seven patients*.

b *Data missing on two patients*.

c *Data missing on four patients*.

d *Data missing on three patients*.

e *Data missing on two patients*.

f *Data missing on one patient*.

Utilizing the HCTS classification, the first two head CT scan reviewers reached a substantial agreement in terms of subdural hematoma, intracerebral hematoma, mass lesions (size > 25 cm^3^), and intraventricular hemorrhage, whereas the agreement was moderate in terms of epidural hematoma and suprasellar cistern features as assessed according to Cohen (29) (Supplementary Table 2). The overall agreement reliability between the reviewers RR, JP, and TL was excellent in terms of subdural hematoma, intracerebral hematoma, mass lesions (size > 25 cm^3^), and intraventricular hemorrhage, whereas the agreement reliability was good in terms of epidural hematoma and suprasellar cistern features as assessed according to Koo and Li (30) (Supplementary Table 3).

The blood samples of all the patients were obtained within 24 h from admission. In those patients for whom the exact time of injury was available, the time elapse from injury to blood sampling was 13.1 ± 10.4 h (*n* = 62). Among those patients in whom the exact injury time was unavailable, the time of injury was estimated based on the best available information. Among these patients, 26 patients were sampled within 24 h, and 49 patients were sampled after 24 h from the injury.

The biomarker levels in different outcome groups are presented in Supplementary Tables 4, 5.

### Helsinki Computed Tomography Scale Alone in Outcome Prediction

The HCTS alone yielded a pAUC of the ROC of 2.5% (1.1–4.7) with a sensitivity of 97.0% (95% CI 90.9–100) and a specificity of 22.4% (95% CI 10.2–32.7) in detecting patients with unfavorable outcome. The threshold to detect a patient with unfavorable outcome was an HCTS sum of >1 (Table 3). In terms of discriminating patients with complete recovery and incomplete recovery, the HCTS did not reach clinically relevant sensitivity and specificity (Table 4).

**Table 3:** Individual abilities of the Helsinki Computed Tomography Score and eight different biomarkers in discriminating patients with favorable and unfavorable outcomes sorted by partial area under the curve of the receiver operating characteristic (all, *n* = 82; favorable outcome, *n* = 49; unfavorable outcome, *n* = 33).

**Biomarker**	**Threshold, pg/ml**	**% pAUC (95% CI)**	**% Specificity (95% CI)**	**% Sensitivity (95% CI)**
HCTS	1	2.5 (1.1–4.7)	22.4 (10.2–32.7)	97.0 (90.9–100)
Aβ40	15.1	2.2 (0.9–4.1)	32.7 (18.4–46.9)	90.9 (81.8–100)
Aβ42	7.9	1.0 (0.1–2.7)	18.4 (8.2–30.6)	90.9 (78.8–100)
NF-L	179.6	0.6 (0.0–3.2)	22.4 (12.2–34.7)	90.9 (78.8–100)
H-FABP	56.3	0.6 (0.0–1.5)	6.1 (0.0–14.3)	100 (100–100)
t-tau	56.5	0.5 (0.0–3.0)	24.5 (12.2–36.7)	90.9 (78.8–100)
IL-10	13.9	0.3 (0.0–1.5)	8.2 (2.0–6.1)	93.9 (84.8–100)
S100B	2300.8	0.2 (0.0–1.5)	2.0 (0.0–6.1)	100 (100–100)
GFAP	94.7	0.1 (0.0–2.6)	12.2 (4.1–22.4)	90.9 (81.8–100)

*Threshold indicates a value or level that needs to be exceeded to detect unfavorable outcome. pAUC, partial area under the curve of the receiver operating characteristic; HCTS, Helsinki Computed Tomography Score; Aβ40, β-Amyloid isoform 1–40; Aβ42, β-Amyloid isoform 1–42; GFAP, glial fibrillary acidic protein; H-FABP, heart fatty acid-binding protein; IL-10, interleukin 10; NF-L, neurofilament light; S100B, S100 calcium-binding protein B; t-tau, total tau*.

**Table 4:** Individual abilities of the Helsinki Computerized Tomography Score and eight different biomarkers in discriminating patients with complete and incomplete recovery sorted by partial area under the curve of the receiver operating characteristic (all, *n* = 82; complete recovery, *n* = 10; incomplete recovery, *n* = 72).

**Biomarker**	**Threshold, pg/ml**	**% pAUC (95% CI)**	**% Specificity (95% CI)**	**% Sensitivity (95% CI)**
Aβ40	35.0	2.3 (0.0–5.3)	40.0 (10.0–70.0)	90.3 (83.3–95.8)
NF-L	245.1	1.2 (0.0–4.1)	20.0 (0.0–50.0)	93.1 (86.1–98.6)
Aβ42	32.9	1.2 (0.0–4.1)	20.0 (0.0–50.0)	91.7 (84.7–97.2)
GFAP	113.9	1.0 (0.0–3.2)	22.4 (12.2–34.7)	94.4 (88.9–98.6)
H-FABP	56.4	0.7 (0.0–2.6)	10.0 (0.0–30.0)	97.2 (93.1–100)
HCTS	–	0.4 (0.0–2.3)	0.0 (0.0–0.0)	100 (100–100)
t-tau	–	0.3 (0.0–3.1)	20.0 (0.0–50.0)	90.3 (83.3–95.8)
IL-10	–	0.0 (0.0–0.0)	0.0 (0.0–0.0)	100 (100–100)
S100B	–	0.0 (0.0–1.4)	0.0 (0.0–0.0)	100 (100–100)

*Threshold indicates a level that needs to be exceeded to detect incomplete recovery. pAUC, partial area under the curve of the receiver operating characteristic; HCTS, Helsinki Computerized Tomography Score; Aβ40, β-Amyloid isoform 1–40; Aβ42, β-Amyloid isoform 1–42; GFAP, glial fibrillary acidic protein; H-FABP, heart fatty acid-binding protein; IL-10, interleukin 10; NF-L, neurofilament light; S100B, S100 calcium-binding protein B; t-tau, total tau*.

### Biomarkers Alone in Outcome Prediction

In discriminating patients with favorable and unfavorable outcomes, the three best individually performing biomarkers in outcome prediction were Aβ40, Aβ42, and NF-L (Table 3). Patients with unfavorable outcome had significantly higher levels of Aβ42 (unfavorable outcome: median 21.9 pg/ml, IQR 40.6 pg/ml; favorable outcome: median 16.9 pg/mL, IQR 16.4 pg/ml; *p* = 0.040) and NF-L (unfavorable outcome: median 99.9 pg/ml, IQR 120.0 pg/ml; favorable outcome: median 36.9 pg/ml, IQR 57.6 pg/ml; *p* = 0.001) compared with those with favorable outcome, whereas levels of Aβ40 were not different between the groups (*p* = 0.490).

In terms of discriminating patients with complete and incomplete recovery, the three best individually performing biomarkers in outcome prediction were Aβ40, NF-L, and Aβ42 (Table 4).

Patients with incomplete recovery had significantly higher levels of NF-L (incomplete recovery: median 66.9 pg/ml, IQR 87.0 pg/ml; complete recovery: median 9.2 pg/ml, IQR 13.5 pg/ml; *p* = 0.001) compared with those with complete recovery, whereas levels of Aβ40 and Aβ42 were not different between the groups (*p* = 0.436 and *p* = 0.257, respectively).

### Biomarkers Improve the Outcome Predictive Performance of the Helsinki Computed Tomography Scale

**Figure 2:**
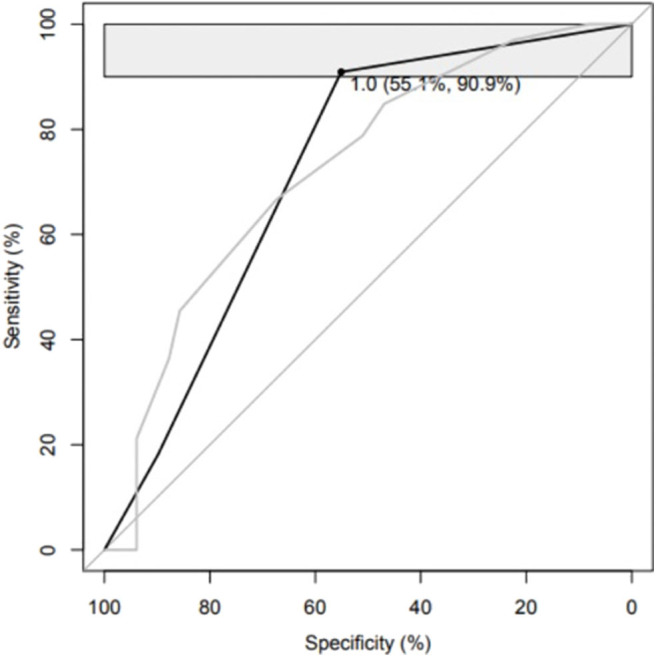
Please see Table 5A.

**Figure 3:**
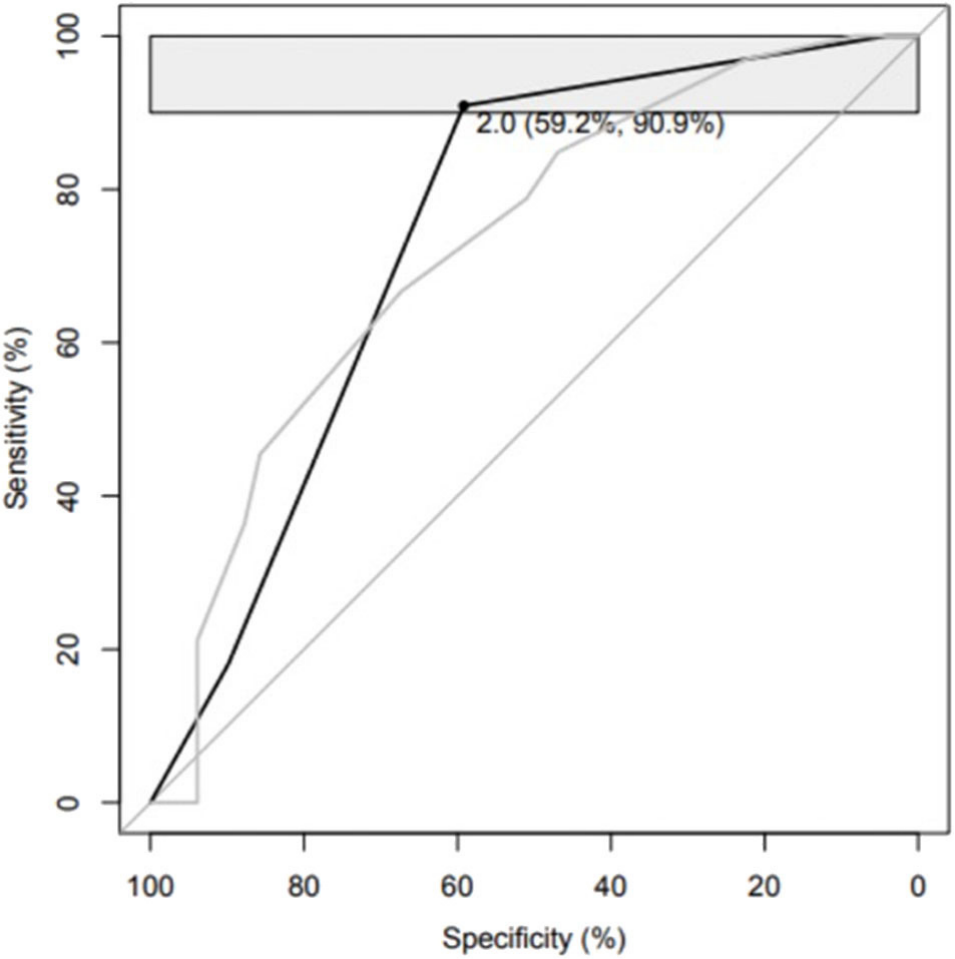
Please see Table 5B.

We studied if combinations of biomarkers could improve the predictive performance of the HCTS in distinguishing patients with unfavorable outcome from patients with a favorable outcome. The best panel consisting of HCTS and a single biomarker included IL-10, and it yielded a pAUC of 3.0% (95% CI 1.3–6.0) with a sensitivity of 90.9% (95% CI 78.8–100) and a specificity of 55.1% (95% CI 40.8–69.4). In this panel, the threshold for the HCTS was >4 and for IL-10 <0.48 mg/ml (Table 5A, Figure 2). A corresponding analysis was conducted with HCTS and a combination of two biomarkers. The optimal panel included IL-10 and Aβ40, and it reached a pAUC of 3.4% (95% CI 1.7–6.2) with a sensitivity of 90.9% (95% CI 81.8–100) and a specificity of 59.2% (95% CI 40.8–69.4). In this panel, the threshold for the HCTS was >4, for Aβ40 >7.38 pg/ml, and for IL-10 <0.48 pg/ml (Table 5B, Figure 3).

**Table 5A:** Ability of the Helsinki Computed Tomography Score alone and a panel consisting of the Helsinki Computed Tomography and interleukin 10 in distinguishing patients with unfavorable outcome from patients with favorable outcome.

	**Markers (threshold to be classified as positive)**	**% pAUC (95% CI)**	**% Specificity (95% CI)**	**% Sensitivity (95% CI)**
HCTS	HCTS (>1)	2.5 (1.2–4.6)	22.4 (12.2–34.7)	97.0 (90.9–100)
Panel	HCTS (>4) + IL-10 (<0.48 pg/ml)	3.0 (1.3–6.0)	55.1 (40.8–69.4)	90.9 (78.8–100)

*Marker thresholds to detect patients with unfavorable outcome are presented in the second column. At least one marker needs to exceed the threshold in order for the panel to be positive. In the figure, a value before the parenthesis indicates that at least one marker needs to be positive (exceed the threshold) in the panel. Values in the parenthesis are the specificity and sensitivity of the panel*.

*HCTS, Helsinki Computerized Tomography Score; IL-10, interleukin 10*.

**Table 5B:** Abilities of the Helsinki Computed Tomography Score alone and a panel consisting of the Helsinki Computed Tomography, interleukin 10, and β-Amyloid isoform 1–40 in distinguishing patients with unfavorable outcome from patients with favorable outcome.

	**Markers (threshold to be classified as positive)**	**% pAUC (95% CI)**	**% Specificity (95% CI)**	**% Sensitivity (95% CI)**
HCTS	HCTS (>1)	2.5 (1.2–4.6)	22.4 (12.2–34.7)	97.0 (90.9–100)
Panel	HCTS (>4) + IL-10 (<0.48 pg/ml) + Aβ40 (>7.38 pg/ml)	3.4 (1.7–6.2)	59.2 (44.9–71.4)	90.9 (78.8–100)

*Marker thresholds to detect patients with unfavorable outcome are presented in the second column. At least two markers need to exceed the threshold in order for the panel to be positive. In the figure, a value before the parenthesis indicates that at least two markers need to be positive (exceed the threshold) in the panel. Values in the parenthesis are the specificity and sensitivity of the panel*.

*HCTS, Helsinki Computerized Tomography Score; IL-10, interleukin 10; Aβ40, β-Amyloid isoform 1–40*.

Panel analysis for outcome prediction of incomplete recovery was not conducted because the HCTS did not have a clinically meaningful outcome prediction performance in this setting (Table 4).

### Biomarkers in Outcome Prediction in Patients With Normal Head Computed Tomography Findings

To further elucidate the outcome prediction performance of the biomarkers, we also studied patients with CT-negative TBIs (comparison study cohort). The three best individually performing biomarkers in discriminating patients with a favorable outcome and an unfavorable outcome were Aβ40, GFAP, and NF-L (Table 6). The three best individually performing biomarkers in discriminating patients with complete and incomplete recovery were NF-L, Aβ40, and IL-10 (Table 7).

**Table 6:** Individual abilities of eight different biomarkers in discriminating patients with favorable and unfavorable outcomes without head imaging abnormalities sorted by partial area under the curve of the receiver operating characteristic (all, *n* = 55; favorable outcome, *n* = 51; unfavorable outcome, *n* = 4).

**Biomarker**	**Threshold, pg/ml**	**% pAUC (95% CI)**	**% Specificity (95% CI)**	**% Sensitivity (95% CI)**
Aβ40	16.7	5.1 (3.7–7.3)	51.0 (37.3–64.7)	100 (100–100)
GFAP	0.4	4.5 (3.3–8.0)	45.1 (31.4–58.8)	100 (100–100)
NF-L	8.3	4.1 (2.7–10.0)	41.2 (27.5–54.9)	100 (100–100)
t-tau	1.6	3.6 (2.4–7.5)	35.3 (21.6–49.0)	100 (100–100)
H-FABP	3.8	3.3 (2.2–8.0)	33.3 (19.6–47.1)	100 (100–100)
IL-10	0.2	2.4 (1.4–9.4)	23.5 (13.7–35.3)	100 (100–100)
S100B	45.3	1.4 (0.6–6.7)	13.7 (5.9–23.5)	100 (100–100)
Aβ42	–	0.0 (0.0–6.5)	0.0 (0.0–0.0)	100 (100–100)

*Threshold indicates a level that needs to be exceeded to detect unfavorable recovery. pAUC, partial area under the curve of the receiver operating characteristic; Aβ40, β-Amyloid isoform 1–40; Aβ42, β-Amyloid isoform 1–42; GFAP, glial fibrillary acidic protein; H-FABP, heart fatty acid-binding protein; IL-10, interleukin 10; NF-, neurofilament light; S100B, S100 calcium-binding protein B; t-tau, total tau*.

**Table 7:** Individual abilities of the eight different biomarkers in discriminating patients with complete and incomplete recovery without head imaging abnormalities sorted by partial area under the curve of the receiver operating characteristic (all, *n* = 55; complete recovery, *n* = 32; incomplete recovery, *n* = 23).

**Biomarker**	**Threshold, pg/ml**	**% pAUC (95% CI)**	**% Specificity (95% CI)**	**% Sensitivity (95% CI)**
NF-L	4.9	0.9 (0.0–2.9)	17.4 (4.3–34.8)	93.8 (84.4–100)
Aβ40	4.3	0.5 (0.0–2.6)	4.3 (0.0–13.0)	100 (100–100)
IL-10	8.0	0.4 (0.0–1.7)	4.3 (0.0–13.0)	100 (100–100)
GFAP	–	0.2 (0.0–1.3)	0.0 (0.0–0.0)	100 (100–100)
H-FABP	–	0.2 (0.0–2.8)	0.0 (0.0–0.0)	100 (100–100)
Aβ42	–	0.0 (0.0–2.4)	0.0 (0.0–0.0)	100 (100–100)
S100B	–	0.0 (0.0–1.1)	0.0 (0.0–0.0)	100 (100–100)
t-tau	–	0.0 (0.0–3.9)	0.0 (0.0–0.0)	100 (100–100)

*Threshold indicates a level that needs to be exceeded to detect incomplete recovery. Statistically significant p-values are in bold. Mann U, Mann–Whitney U-test; pAUC, partial area under the curve of the receiver operating characteristic; Aβ40, β-Amyloid isoform 1–40; Aβ42, β-Amyloid isoform 1–42; GFAP, glial fibrillary acidic protein; H-FABP, heart fatty acid-binding protein; IL-10, interleukin 10; NF-L, neurofilament light; S100B, S100 calcium-binding protein B; t-tau, total tau*.

## Discussion

This prospective, observational study of patients with acute TBI investigated whether admission levels of eight different plasma protein biomarkers obtained from CT-positive patients can improve the outcome prediction ability of the HCTS without clinical covariates in a well-characterized cohort. We also studied the prognostic ability of the biomarkers without the HCTS in discriminating complete recovery and incomplete recovery in CT-positive patients and CT-negative patients. The main finding of the study is that the admission levels of IL-10 and Aβ40 improve the ability of the HCTS in discriminating patients with unfavorable and favorable outcomes with increasing the specificity by 27% points (from 22 to 59%) while maintaining a sensitivity above 90%. In other words, when using only the HCTS, 11 patients out of the 49 with favorable outcomes were correctly detected, and when using the HCTS together with biomarkers, 29 patients with favorable outcomes were correctly detected. When studied alone, the HCTS had the highest pAUCs of the tested covariates, followed by Aβ40 and Aβ42. The individual specificities of the HCTS and biomarkers remained low (2–33%) in isolation, but the optimal combination panel yielded a specificity of 59% when the sensitivity was set above 90%.

Most modern TBI biomarker studies have investigated the individual prediction abilities of different molecules. The studies show that single biomarkers tend to have low specificities when sensitivity is set above 90%. Therefore, individual blood-based biomarkers may not be applicable for clinical practice as stand-alone tools (19, 28, 31), which is expected due to the complexity of TBI. Combining several biomarkers or combining biomarkers with clinical characteristics have been suggested to improve diagnostic and predictive abilities (31, 32). Thus, biomarkers may provide additional value in outcome prediction of TBI when used in combination with predictive neuroimaging scores. However, studies on blood-based biomarkers complementing head imaging scores are scarce. The results presented here suggest that protein biomarkers IL-10 and Aβ40 provide incremental value in outcome prediction when used in combination with the HCTS. Intriguingly, in both panels in the panel analysis, the thresholds for IL-10 (many patients with lower GCS scores—indicating a more severe TBI—have relatively low levels of IL-10) and Aβ40 are considerably lower and for the HCTS higher compared to analyses where the parameters are studied in isolation. In line with this finding, it has been previously reported that most of the clinical studies have not identified a correlation between blood IL-10 levels and GCS scores (33). These results suggest that the best diagnostic value in discriminating patient outcomes after TBI is achieved by utilizing biomarkers in combination, which echoes our other recent findings in the acute diagnostics of TBI (19) and outcome prediction (34). A possible explanation for the higher HCTS threshold in the panel analysis is that biomarkers provide additional accuracy to the predictive power of the HCTS permitting patients with a favorable outcome to have some traumatic intracranial findings. We have recently reported IL-10 thresholds of 0.38 and 0.44 pg/ml depending on other markers included in the panels for predicting unfavorable outcomes. Correspondingly, when the HCTS is included in the panels, IL-10 thresholds need to be lower to capture patients with low IL-10 levels, low GCS scores, and unfavorable outcomes.

To better illuminate the predictive power of biomarkers in patients with CT-positive findings, we also investigated their abilities in distinguishing between patients with complete and incomplete recovery. The best-performing biomarkers were the same as in discrimination of patients with favorable and unfavorable outcomes, but the predictive performance of the HCTS was low. The HCTS was designed to predict functional outcome according to the GOS (13). Thus, unsurprisingly, the HCTS does not provide enough information to clinically meaningfully discriminate between patients with complete and incomplete recovery.

We also conducted a comparative analysis of CT-negative patients. In discriminating CT-negative patients with favorable and unfavorable outcomes, the best performing biomarkers were Aβ40, GFAP, and NF-L. However, these results should be interpreted with caution due to the small number of patients with unfavorable outcomes among CT-negative patients. In predicting a full recovery in CT-negative patients, only NF-L, Aβ40, and IL-10 showed a modest predictive power, whereas the other proteins did not have any prognostic value.

We utilized the pAUC instead of the conventional AUC test. The AUC indexes diagnostic performance summarizing the entire ROC curve, including regions that might not be relevant to a certain clinical application (e.g., regions with low levels of sensitivity or specificity). To overcome this disadvantage, we used the pAUC that summarizes a portion of the ROC curve over the prespecified range of interest (35)—in the context of the current study, sensitivity >90%. Thus, the pAUC yields more information regarding the predictive information provided by the HCTS and biomarkers than, for example, overall median value comparison using the Mann–Whitney *U-*test. This explains the finding that median levels of Aβ40 were not different between the favorable and unfavorable outcome groups, but the biomarkers still yield a good pAUC and specificity when studied in panels within a fixed sensitivity area. This also applies to the finding why Aβ40 and Aβ42 are not different between the complete and incomplete recovery groups.

Clinical features are known to contribute to explaining outcome variance (3). However, given the primary purpose of the current analysis was to explore the prognostic and diagnostic performance of the biomarker studied as an adjunct to CT imaging, they were not integrated into the overall prognostic models. In the main study cohort, there were no differences in sex distribution, pupil reactivity, events of hypoxia, events of hypotension, hypoglycemia, anemia, and the proportion of hospital admissions. Extracranial injuries may affect the levels of GFAP, H-FABP, IL-10, NF-L, S100B, and t-tau (18, 19, 26), but in terms of patient group comparisons in the main study cohort, this effect can be considered negligible because the proportion of patients with concomitant extracranial injuries was similar. Moreover, we have previously demonstrated that the levels of IL-10 and Aβ40—the proteins included in the outcome prediction panels in the current study—are not affected by the presence of extracranial injuries in patients with TBIs of all severities and CT-positive findings (19). The differences in the HCTS features reflect more serious lesion load in patients with unfavorable outcomes. The patients were also older in the unfavorable outcome group.

We studied several biomarkers that are known to be correlated with TBI prognosis, but we also selected biomarkers less investigated in the literature due to their recent promising results in acute TBI diagnostics (19, 36, 37). Astroglial marker S100B is the most studied TBI biomarker to date (38–40). Acutely (12–36 h) measured blood S100B levels are associated with outcome (41). An earlier study reported that levels of S100B and GFAP in combination are correlated with unfavorable outcome in patients with severe TBI (42). S100B is expressed in many bodily tissues outside the central nervous system, and its levels increase, e.g., after extracranial injuries (43) and physical exercise (44), which may complicate interpretation of the results if the patient has significant extracranial injuries and if the levels are assessed in polytrauma patients immediately after injury (45, 46). After S100B, the astroglial marker GFAP, which is expressed in the cytoskeleton of glial cells (47), is probably the most studied TBI biomarker. Many studies have shown a significant association between increased GFAP levels and unfavorable outcome (16, 17, 42, 48). NF-L and tau have been mostly studied in the subacute after TBI. NF-L is abundantly expressed in the long myelinated subcortical axons (49). NF-L has been reported to be significantly correlated with late outcome after TBI by three studies (17, 50, 51). Tau is a microtubule-associated protein expressed in the axonal cytoskeleton (52, 53). Significant increases in tau levels have been reported in concussed professional ice hockey players (54), and tau levels have been correlated with outcome after severe TBI (55). Aβ40 and Aβ42 (52, 56) are associated with amyloidogenic amyloid precursor protein metabolism and have been suggested as potential biomarkers of axonal damage in TBI (57). However, it has been reported that especially in the case of mild TBI, Aβ40 and Aβ42 do not exhibit prognostic value (58–60). Cytosolic trafficking protein H-FABP and anti-inflammatory mediator protein IL-10 are related to traumatic intracranial findings (19, 36, 37). The outcome prediction ability of IL-10 after TBI has been controversial, although it has shown some potential in predicting mortality (33). However, a recent study utilizing partially same cohort as in this study demonstrated that both IL-10 and H-FABP improved outcome prediction abilities of panels consisting of more studied biomarkers and clinical covariates in both mild TBI and TBIs of all severities (34).

Previous studies suggest that biomarkers may perform in the outcome prediction of TBI better in combination than in isolation (50, 61, 62). Czeiter et al. (63) have reported that GFAP has an added value when combined with a modified IMPACT model consisting of age, GCS motor score, and pupil status. Both Gradisek and Vos have reported that GFAP and S100B improve the performance of clinical parameters in outcome prediction (61, 62). These findings are consistent with a recent study by Thelin et al. (18), where they reported that GFAP and NF-L enhanced the predictive ability of the IMPACT model combined with the Stockholm CT findings. With regard to current results, there was no benefit to combining HCTS, GFAP, and S100B with HCTS.

Currently, the most widely used CT scores are the Marshall CT classification and Rotterdam CT score. The Marshall CT classification grades injuries—in non-ordinal fashion—as different levels of diffuse injuries or mass lesions in case hematoma volume exceeds 25 cm^3^ (8). Although the Marshall CT classification was not designed to be used as an outcome prediction tool, the Rotterdam CT score was developed based on the Marshall CT classification features adding traumatic subarachnoid and intraventricular hemorrhage (12). The most recent additions to the outcome prediction-weighted CT classifications are the HCTS and Stockholm CT score. The Stockholm CT score includes a separate traumatic subarachnoid hemorrhage score and a tally comprising midline shift as a continuous variable, epidural hematoma, dual-sided subdural hematoma, diffuse axonal injury, and the traumatic subarachnoid hemorrhage score (14). The HCTS focuses on the types of intracranial gross pathologies (13). It has been reported that the Stockholm CT score and HCTS outperform the older scores in outcome prediction (15). We chose the HCTS because its implementation is reliable, it is widely validated, and it takes into account different types of intracranial injuries that may be associated with differently elevated biomarker levels.

The strengths of this study are the use of several biomarkers of different cellular origins in the same cohort, the use of sensitive advanced analytics, and a prospectively recruited well-characterized study population. Although a minority of the screened patients were included in the current analyses, the patient selection did not introduce a significant bias, as the only difference was sex distribution.

The main limitation of the study is the variable delays between injuries and blood sampling. This may have affected biomarkers with a short half-life in blood, such as H-FABP, IL-10, and S100B. Furthermore, for NF-L, the sampling time-points might have been too close to the injury (64). Earlier mean sampling time would probably have resulted in different sensitivities and specificities for the panels. In addition, we could not use the levels of UCH-L1 from the Human Neurology 4-Plex assay in the current analyses because the coefficients of variation were at a level where the results are not reliable. We also used the National Institute for Health and Care Excellence criteria for head CT imaging, and the results might not be applicable for other head CT rules due to differences in case selection. The fairly small study cohort also increases the risk of over-fitting bias, and therefore, the results should be verified and validated in a larger cohort. Moreover, the assays utilized in this study are developed for research purposes, limiting the generalizability of the results. However, this limitation also concerns most of the current TBI biomarker studies because there is a paucity of commercialized assays for clinical TBI diagnostics. The possibility of some degree of selection bias should be noted, as only a third of the patients treated at the recruiting hospital were eventually enrolled in the study. The current cohort is somewhat less severely injured than those in which the HCTS has been earlier validated. The HCTS was originally designed using a neurocritical care cohort. Finally, these results specifically speak to the additional ability of the biomarkers studied to improve on the ability of the Helsinki CT Score to explain outcome variance. Integration into well-established TBI outcome prediction schemes such as IMPACT (9) and Corticosteroid Randomization After Significant Head injury (10) will require further study. The authors acknowledge the limitations of the GOSE in detecting subtle functional and cognitive deficits, especially in patients with higher GOSE scores. However, the main aim of the study was slightly grosser in terms of prognostication, as we studied whether different protein biomarkers can improve the outcome prediction performance of the HCTS in discriminating patients with favorable and unfavorable outcomes. The variability in the time interval between injury and GOSE assessment may have affected the results.

## Conclusion

Admission levels of IL-10 and Aβ40 improve the prognostic performance of the HCTS in discriminating patients with unfavorable and favorable outcomes. When studied alone, HCTS had the highest pAUCs of the tested covariates, followed by Aβ40 and Aβ42. Although the individual specificities of the HCTS and biomarkers remained low (2–33%) in isolation, the optimal combination panel yielded a specificity of 59% when the sensitivity was set above 90%. The current results suggest that outcome prediction ability of the HCTS could be significantly enhanced with rapid point-of-care measurement of plasma levels of IL-10 and Aβ40. This may allow the identification of initially neurologically stable patients who, however, are developing severe secondary brain injury that significantly impairs their recovery.

## Data Availability Statement

The datasets generated for this study are available on request to the corresponding author.

## Ethics Statement

The studies involving human participants were reviewed and approved by the ethical review board of the Hospital District of Southwest Finland. The patients/participants provided their written informed consent to participate in this study.

## Author Contributions

JP, RT, RR, and TL conceived and designed the current study. JP, RT, AK, H-RM, JT, and OT recruited the patients. JP, RT, AK, MM, IH, H-RM, JT, PK, and OT collected and curated the data. LA, LL, J-CS, and JP conducted the statistical analyses. JG, HZ, KB, and J-CS supervised the biomarker analyses. MG, PH, DM, VN, and OT supervised the TBIcare study. JP drafted the manuscript with critical contributions from RT, RR, and TL. JP takes the responsibility for the paper as a whole. All authors substantially contributed to the revision of the manuscript.

## Conflict of Interest

JP has received a speaker's fee from the Finnish Medical Association. KB has served as a consultant or at advisory boards for Abcam, Axon, Biogen, Lilly, MagQu, Novartis, and Roche Diagnostics and is a co-founder of Brain Biomarker Solutions in Gothenburg AB, a GU Venture-based platform company at the University of Gothenburg. The remaining authors declare that the research was conducted in the absence of any commercial or financial relationships that could be construed as a potential conflict of interest. The handling editor is currently organizing a Research Topic with some of the authors OT and J-CS.
