# Supplementary material for: Admission Levels of Interleukin 10 and Amyloid β 1–40 Improve the Outcome Prediction Performance of the Helsinki Computed Tomography Score in Traumatic Brain Injury
Source: Front Neurol. 2020 Oct 30;11:549527. doi: 10.3389/fneur.2020.549527 (PMC7661930; doi:10.3389/fneur.2020.549527)
Supplement: Supplementary file 1 [file Data_Sheet_1.PDF]

Supplementary table 1. Biomarkers, assays used and assay properties

Biomarker	Assay	LLoD	LLoQ	Calibration range
Aβ40	Neurology Duplex (Quanterix)	0.045 pg/mL	0.142 pg/mL	0–90.0 pg/mL
Aβ42	Neurology Duplex (Quanterix)	0.142 pg/mL	0.69 pg/mL	0–11.0 pg/mL
GFAP	Neurology 4-Plex (Quanterix)	0.221 pg/mL	0.467 pg/mL	0.934–891 pg/mL
H-FABP	K151HTD (Meso Scale)	0.103 ng/mL	Not available*	0.137–100 ng/mL
IL-10	K151QUD (Meso Scale)	0.04 pg/mL	0.298 pg/mL	0.0774–327 pg/mL
NF-L	Neurology 4-Plex (Quanterix)	0.104 pg/mL	0.241 pg/mL	0.481–500 pg/mL
S100B	EZHS100B-33K (Millipore)	2.7 pg/mL	Not available*	2.7–2000 pg/mL
t-tau	Neurology 4-Plex (Quanterix)	0.024 pg/mL	0.053 pg/mL	0.105–94.3 pg/mL

LLoD, lower level of detection; LLoQ, lower level of quantification; Aβ40, β-Amyloid isoform 1-40; Aβ42 β-Amyloid isoform 1-42; GFAP, glial fibrillary acidic protein; H-FABP, heart fatty acid binding protein; IL-10, interleukin 10; NF-L neurofilament light; S100B, S100 calcium-binding protein B; ; t-tau, total tau; *No established limit

Supplementary table 2. The first round of the head computed tomography scan review: interrater reliability for independent scan reviewers #1 and #2.

HCTS features			Reviewer #2		Cohen's κ
Mass lesion types			No subdural hematoma (n)	Subdural hematoma (n)	
	Reviewer #1	No subdural hematoma (n)	79	5	0.732, **p<0.001**
		Subdural hematoma (n)	12	41	
			No intracerebral hematoma (n)	Intracerebral hematoma (n)	
	Reviewer #1	No intracerebral hematoma (n)	66	4	0.667, **p<0.001**
		Intracerebral hematoma (n)	18	49	
			No epidural hematoma (n)	Epidural hematoma (n)	
	Reviewer #1	No epidural hematoma (n)	124	3	0.437, **p<0.001**
		Epidural hematoma (n)	6	4	
Mass lesion size >25 cm^3			No mass lesion size >25 cm^3 (n)	Mass lesion size >25 cm^3 (n)	
	Reviewer #1	No mass lesion size >25 cm^3 (n)	109	4	0.769, **p<0.001**
		Mass lesion size >25 cm^3 (n)	5	19	
Intraventricular hemorrhage			No intraventricular hemorrhage (n)	Intraventricular hemorrhage (n)	
	Reviewer #1	No intraventricular hemorrhage (n)	114	3	0.791, **p<0.001**
		Intraventricular hemorrhage (n)	4	16	
Supracellar cisterns			No compressed supracellar cisterns (n)	Compressed supracellar cisterns (n)	
	Reviewer #1	No compressed supracellar cisterns (n)	99	4	0.501, **p<0.001**
		Compressed supracellar cisterns (n)	18	16	
			No obliterated supracellar cisterns (n)	Obliterated supracellar cisterns (n)	
	Reviewer #1	No obliterated supracellar cisterns (n)	127	1	0.439, **p<0.001**
		Obliterated supracellar cisterns (n)	6	3	

Statistically significant p values are in bold; Cohen's κ, Cohen's kappa

Supplementary table 3. The whole head computed tomography scan review process: interrater reliability between independent scan reviewers #1, #2 and #3.

HCTS features		Intraclass correlation coefficient	95%CI	p value
Mass lesion types	Subdural hematoma	0.933	0.911–0.951	**<0.001**
	Intracerebral hematoma	0.915	0.885–0.938	**<0.001**
	Epidural hematoma	0.852	0.803–890	**<0.001**
Mass lesion size >25 cm^3		0.945	0.927–0.959	**<0.001**
Intraventricular hemorrhage		0.950	0.934–0.963	**<0.001**
Supracellar cisterns	Compressed supracellar cisterns	0.863	0.816–0.899	**<0.001**
	Obliterated supracellar cisterns	0.802	0.736–0.853	**<0.001**

Statistically significant p values are in bold

Supplementary table 4. Biomarker levels and their differences in computed tomography-positive patients with favorable and unfavorable outcome

Biomarker	Favorable outcome (n=49) median [interquartile range (range)]	Unfavorable outcome (n=33) median [interquartile range (range)]	Mann-Whitney U, p value
Aβ40	22.6 [13.0 (6.4–52.7)]	24.8 [21.3 (3.4–56.0)]	0.490
Aβ42	16.9 [16.4 (3.0–52.7)]	21.9 [13.2 (5.0–38.1)]	**0.040**
GFAP	6.0 [15.2 (0.07–109.2)]	37.9 [79.7 (0.1–1763.1)]	**0.001**
H-FABP	6.7 [11.54 (1.7–58.4)]	11.5 [40.6 (2.1–60.9)]	**0.019**
IL-10	0.8 [1.1 (0.2–33.6)]	1.5 [5.3 (0.4–66.2)]	**0.003**
NF-L	36.9 [57.6 (6.7–1298.0)]	99.9 [120.0 (5.7–1422.2)]	**0.001**
S100B	75.8 [88.5 (9.4–545.8)]	215.3 [487.7 (10.4–2323.8)]	**0.001**
t-tau	4.2 [25.5 (0.3–116.1)]	29.2 [47.4 (0.7–1186.0)]	**0.001**

All biomarker levels are presented in pg/mL except H-FABP, which is in ng/mL. Statistically significant p values are in bold; Aβ40, β-Amyloid isoform 1-40; Aβ42 β-Amyloid isoform 1-42; GFAP, glial fibrillary acidic protein; H-FABP, heart fatty-acid binding protein; IL-10, interleukin 10; NF-L neurofilament light; S100B, S100 calcium-binding protein B; t-tau, total tau

Supplementary table 5. Biomarker levels and their differences in computed tomography-positive patients with complete and incomplete recovery

Biomarker	Complete recovery (n=10) median [interquartile range (range)]	Incomplete recovery (n=72) median [interquartile range (range)]	Mann-Whitney U, p value
Aβ40	19.4 [15.9 (8.6–35.6)]	24.2 [16.0 (3.4–55.6)]	0.436
Aβ42	17.1 [16.7 (3.0–27.6)]	20.7 [16.9 (3.0–39.2)]	0.257
GFAP	1.7 [5.9 (0.1–109.2)]	13.7 [49.8 (0.07–1763.1)]	**0.032**
H-FABP	4.7 [2.6 (2.8–58.4)]	8.8 [22.0 (1.7–60.9)]	**0.020**
IL-10	0.3 [1.2 (0.2–5.8)]	1.0 [2.4 (0.2–66.2)]	**0.019**
NF-L	9.2 [13.5 (6.8–1182.6)]	66.9 [87.0 (5.7–1422.2)]	**0.001**
S100B	73.0 [47.5 (12.2–198.4)]	113.8 [22.0 (9.4–2323.8)]	0.061
t-tau	2.3 [7.2 (1.2–40.4)]	18.3 [44.4 (0.3–1186.0)]	**0.019**

All biomarker levels are presented in pg/mL except H-FABP, which is in ng/mL. Statistically significant p values are in bold; Aβ40, β-Amyloid isoform 1-40; Aβ42 β-Amyloid isoform 1-42; GFAP, glial fibrillary acidic protein; H-FABP, heart fatty-acid binding protein; IL-10, interleukin 10; NF-L neurofilament light; S100B, S100 calcium-binding protein B; t-tau, total tau
